# 

**Published:** 1890-11

**Authors:** 


THE seventh edition of "Da Costa's Medical Diagnosis" is now announced by J. B. Lippincott Company as ready. The work has undergone a thorough revision at the hands of its eminent author, and many chapters have been entirely re-written, so as to inculcate all that has been added to our knowledge of disease up to the present time. A number of wood-cuts are included, especially of such microörganisms as have proved to be of practical significance in diagnosis. All the illustrations are original, and many are from sketches, or based on sketches, taken directly from cases of interest. There is no work more helpful to a young practitioner than this one, which has already been pronounced by eminent critics "the best book on diagnosis extant."

---

ANOTHER valuable book just issued by J. B. Lippincott Company, is Prof. Garretson's Treatise on the Diseases and Surgery of the Mouth, Jaws, Face, Teeth, and Associate Parts. Upon the appearance of the first edition many years ago, it assumed the leading place as a text-book, to which its merit and the distinguished position of its author entitled it. Much important matter has been added to the new edition, together with numerous illustrations, which greatly increase its value to dentists, surgeons, and physicians.

---

MRS. GRANT'S LITERARY WORK.—Mrs. Ulysses S. Grant has been induced by a New York editor to tell the story of her courtship with General Grant, and the warrior's proposal to her, in an article published in the October number of *The Ladies' Home Journal.*

---

CAMPANINI, the famous tenor, has written a striking article on "How to Train the Voice," for *The Ladies' Home Journal*, and it will appear in the November number of that periodical.

The Southern Surgical and Gynecological Association will hold its regular annual meeting at Atlanta, Ga., Tuesday, Wednesday, and Thursday, November 11, 12, and 13, 1890, under the presidency of

Dr. George J. Engelmann, of St. Louis. The permanent secretary, Dr. W. E. B. Davis, of Birmingham, Ala., is out with the preliminary programme that contains a long list of valuable papers.

---

The Medical Society of the State of New York. — The eighty-fifth annual meeting will be held in the City of Albany, Tuesday, Wednesday, and Thursday, February 3, 4, and 5, 1891. The Business Committee has been appointed, and is composed of the following-named gentlemen: Dr. Herman Bendell, 178 State street, Albany, *Chairman;* Dr. Seneca D. Powell, 12 West 40th street, New York; and Dr. James D. Spencer, Watertown.

It is desirable that those who intend to present papers should notify one of the members of the Business Committee, giving titles, on or before December 15, 1890, as the programme will be made up and issued *early* in January.

William Warren Potter,
*President.*

284 Franklin Street, Buffalo, N. Y., October 6, 1890.

---

The Microscopical Club will hold its next meeting Tuesday, November 11, 1890, with the following programme: Exhibition, W. H. Bergtold, M. D.; Some Points in Photomicrography, George H. Blackham, M. D., F. R. M. S., Dunkirk, N. Y.; Street Car Straps, Lucien Howe, M. R. C. S. (Eng.)

---

The Buffalo Pathological Society will hold its next meeting Friday, November 21, 1890, at eight o'clock P. M. Annual address of the President, Dr. DeLancey Rochester. Exhibition of pathological specimens.

---

The Buffalo Obstetrical Society's next meeting will be held Tuesday, November 25, 1890, at 8.30 P. M. Essayist, Dr. Banta. Subject, The After-coming Head.

---

The Buffalo Medical and Surgical Association will hold its next meeting Tuesday, November 4, 1890, at 8.15 P. M.

### BOOKS AND PAMPHLETS RECEIVED.

A Text-Book of Comparative Physiology, for Students and Practitioners of Comparative (Veterinary) Medicine. By Wesley Mills, M. A., M. D., D. V. S., Professor of Physiology in the Faculty of Human Medicine and the Faculty of Comparative Medicine and Veterinary Science of McGill University, Montreal; author of a text-book of Animal Physiology, etc. With 476 illustrations. Small octavo; pp. xx.—636. New York: D. Appleton & Co. 1890.

The Biography of Ephraim McDowell, M. D., "The Father of Ovariotomy." By his Granddaughter, Mary Young Ridenbaugh. Together with valuable scientific treatises and articles relating to Ovariotomy, and eulogistic letters from eminent members of the medical profession in Europe and America. Small octavo, full Russia, gilt; pp. xvi.—538. New York: Charles L. Webster & Company. 1890. Wm. J. Dornan, Philadelphia, Printer.

Medical Diagnosis, with Special Reference to Practical Medicine. A Guide to the Knowledge and Discrimination of Diseases. By J. M. DaCosta, M. D., L. L. D., Professor of Practice of Medicine and Clinical Medicine at the Jefferson Medical College, Philadelphia; Physician to the Pennsylvania Hospital; Consulting Physician to the Children's Hospital, etc., etc. Illustrated with engravings on wood. Seventh edition, revised. 8vo; pp. 995. Philadelphia: J. B. Lippincott Company. London: 10 Henrietta street, Covent Garden. 1890. Price, cloth, $6.00.

A Manual of Modern Surgery. An Exposition of the Accepted Doctrines and Approved Operative Procedures of the Present Time. For the use of students and practitioners. By John B. Roberts, A. M.,

M. D., Professor of Surgery in the Woman's Medical College of Pennsylvania; Professor of Anatomy and Surgery in the Philadelphia Polyclinic; Lecturer in Anatomy in the University of Pennsylvania. Octavo, 780 pages, 501 illustrations. Cloth, $4.50; leather, $5.50. Philadelphia: Lea Brothers & Co. 1890.

Transactions of the American Surgical Association. Volume the Eighth. Edited by J. Ewing Mears, M. D., Recorder of the Associaciation. 8vo; pp. xx.—290. Philadelphia: Printed for the Association and for sale by P. Blakiston, Son & Co. 1890. Dornan, Printer.

Essentials of Practice of Medicine. Arranged in the form of Questions and Answers. Prepared especially for students of medicine. By Henry Morris, M. D., late Demonstrator Jefferson Medical College; Visiting Physician to St. Joseph's Hospital, etc. With a very complete appendix on the Examination of Urine. By Lawrence Wolff, M. D., Demonstrator of Chemistry, Jefferson Medical College. Saunders's Question Compends, Nos. 8 and 9. Double Number. Philadelphia: W B. Saunders. 1890.

Ointments and Oleates, Especially in Diseases of the Skin. By John V. Shoemaker, A. M., M. D., Professor of Materia Medica, Pharmacology, and Clinical Medicine, and Clinical Professor of Diseases of the Skin in the Medico-Chirurgical College of Philadelphia; Physician to the Medico-Chirurgical Hospital, etc. Second edition, revised and enlarged. No. 6 in the Physician's and Student's Ready Reference Series. Philadelphia and London: F. A. Davis. 1890.

Epilepsy: Its Pathology and Treatment. Being the Essay to which was awarded a prize of 4,000 francs by the Academie Royale de Médécine de Belgique, December 31, 1889. By Hobart Amory Hare, M. D., B. Sc., Clinical Professor of the Diseases of Children and Demonstrator of Therapeutics in the University of Pennsylvania; Physician to St. Agnes' Hospital and to the Children's Dispensary of the Children's Hospital, etc. No. 7 in the Physician's and Student's Ready Reference Series. Philadelphia and London: F. A. Davis. 1890.

Essentials of the Diseases of Children. Arranged in the form of questions and answers, prepared especially for Students of Medicine. By William M. Powell, M. D., Physician to the Clinic for the Diseases of Children in the Hospital of the University of Pennsylvania, etc. Saunders's Question Compends, No. 15. Philadelphia: W. B. Saunders. 1890.

Transactions of the Medical Society of the State of Pennsylvania, at its fortieth annual session, held at Pittsburg, 1889-90. Volume XXI. Published by the Society. Philadelphia: Wm. J. Dornan, Printer. 1890.

The Science and Art of Obstetrics. By Theophilus Parvin, M. D., LL. D., Professor of Obstetrics and Diseases of Women and Children in Jefferson Medical College, Philadelphia, and one of the Obstetricians to the Philadelphia Hospital. Second edition, revised and enlarged. Illustrated with 231 wood cuts and a colored plate. Pp. xvi.—704. Philadelphia: Lea Brothers & Co. 1890. Price, cloth, $4.25; leather, $5.25. Buffalo: Peter Paul & Bro.

A Treatise on the Diseases of Infancy and Childhood. By J. Lewis Smith, M. D., Clinical Professor of Diseases of Children in Bellevue Hospital Medical College, New York, etc., etc. Seventh edition, thoroughly revised. In one octavo volume of 881 pages, with 51 illustrations. Cloth, $4.50; leather, $5.50; Philadelphia: Lea Brothers & Co. 1890. Buffalo: Peter Paul & Bro.

A Dictionary of Practical Medicine. By various writers. Edited by J. Kingston Fowler, M. A., M. D., Fellow of the Royal College of Physicians; Senior Assistant to the Middlesex Hospital, and Lecturer on Pathological Anatomy in the Medical School; Senior Assistant Physician to the Hospital for Consumption and Diseases of the Chest. Brompton. 8vo; pp. xxvi.—942. Philadelphia: P. Blakiston, Son & Co. 1890. Buffalo: Peter Paul & Bro.

The Medical Student's Manual of Chemistry. By R. A. Witthaus, A. M., M. D., Professor of Chemistry and Physics in the University of the City of New York; Professor of Chemistry and Toxicology in the University of Vermont, Member of the Chemical Societies of Paris and Berlin, Member of the American Chemical Society, Fellow of the American Academy of Medicine, of the New York Academy of Medicine, of the American Association for the Advancement of Science, etc. Third edition. New York: William Wood & Co. 1890. Buffalo: Peter Paul & Bro.

The Essentials of Medical Chemistry and Urinalysis. By Sam. E. Woody, A. M., M. D., Professor of Chemistry and Public Hygiene, and Clinical Lecturer on Diseases of Children in the Kentucky School of Medicine. Third edition. Revised, enlarged, and illustrated. Philadelphia: P. Blakiston, Son & Co., 1012 Walnut street. 1890. Buffalo: Peter Paul & Bro.

A Compend of Surgery. For students and physicians. By Orville Horwitz, B. S., M. D., Demonstrator of Anatomy in Jefferson Medical College; Chief of the Out-door Surgical Department of Jefferson Medical College Hospital, and late Resident Surgeon of the Pennsylvania Hospital, Philadelphia. Quiz Compends No. 9. Third edition; thoroughly revised, enlarged and improved, with 91 illustrations. Philadelphia: P. Blakiston, Son & Co. 1888. Buffalo: Peter Paul & Bro.

A Compend of Equine Anatomy and Physiology. By William B. Ballou, M. D., Professor of Equine Anatomy, and formerly Lecturer on Physiology, New York College of Veterinary Surgeons. Instructor in Genito-Urinary Surgery, New York Polyclinic; Surgeon to Bellevue

Dispensary ; late House-Surgeon, Bellevue Hospital, New York. With twenty-nine graphic illustrations, selected from Chauveau's Comparative Anatomy. Quiz Compends No. 12. Philadelphia: P. Blakiston, Son & Co. 1890. Buffalo : Peter Paul & Bro.

Dust and Its Dangers. By T. Mitchell Prudden, M. D., Author of "A Manual of Practical Normal Histology," "The Story of the Bacteria," etc. New York: G. P. Putnam's Sons. 1890. The Knickerbocker Press. Buffalo : Peter Paul and Bro.

Lectures on Massage and Electricity in the Treatment of Disease (Masso-Electro-Therapeutics). By Thomas Stretch Dowse, M. D., Fellow of the College of Physicians of Edinburgh ; Associate Member of the Neurological Society of New York, etc. Small 8vo ; pp. xx.—379. Medical Classics. New York : E. B. Treat & Company, 5 Cooper Union. Price, $2.75.

Intestinal Diseases of Children. By A. Jacobi, M. D., Clinical Professor of Diseases of Children in the College of Physicians and Surgeons ; ex-President of the New York Academy of Medicine, etc. Two volumes. Second edition. Physician's Leisure Library, Number 6. Detroit: George S. Davis. 1890.

A Practical Treatise on Headache, Neuralgia, Sleep and its Derangements, and Spinal Irritation. By J. Leonard Corning, M. A., M. D., Consultant in Nervous Diseases at St. Francis Hospital ; Fellow of the New York Academy of Medicine ; Member of the New York Neurological Society, etc. Second edition, with an appendix — Eye-Strain, a Cause of Headache. By David Webster, M. D., Professor of Ophthalmology in the New York Polyclinic ; Surgeon to the Manhattan Eye and Ear Hospital, etc., etc. In one large 8vo volume, nearly 300 pages. Price, $2.75. Uniform in style with Medical Classics. Special rate for the set. E. B. Treat, publisher, 5 Cooper Union, New York.

Physical Diagnosis and Practical Urinalysis. An Epitome of the Physical Signs of the Heart, Lung, Liver, Kidney, and Spleen in Health and Disease. Edited by John E. Clark, M, D., Professor of General Chemistry and Physics in the Detroit College of Medicine. Fully illustrated. Cloth, 12mo ; pp. 193. Detroit: *The Illustrated Medical Journal Company*. 1890.

Stricture of the Rectum. A Study of Ninty-six Cases. By Chas. B. Kelsey, M. D., Professor of Diseases of the Rectum at the New York Post-Graduate School and Hospital ; late Professor of Rectal Surgery at the University of Vermont, etc., etc. Paper, 8vo ; pp. 46.

Transactions of the Association of American Physicians. Fifth Session, held at Washington, D. C., May 13, 14, and 15, 1890. Volume V. Cloth, 8vo ; pp. 275. Philadelphia : Printed for the Association, 1890.

Menstruation, and the Removal of Both Ovaries. By George J. Engelmann, A. M., M. D., St. Louis, Mo. Philadelphia: Reprint from Transactions Southern Surgical and Gynecological Association. September, 1889.

The Sewerage of Columbus, Ohio. Address of Col. George E. Waring, Jr., at Board of Trade auditorium, Columbus, O., and Discussion following. The Nestbote Co. 1890.

Extra-Uterine Pregnancy. By E. P. Bernardy, M. D. Reprint from *Annals of Gynecology and Pediatry.* July, 1890.

The Recurrence of Puerperal Fever. By Eugene P. Bernardy, M. D. Philadelphia. Paper, pp. 6.

Binoxide of Mercury. Its Antiseptic Use. By Eugene P. Bernardy, M. D. Philadelphia. Reprint from Transactions of the Philadelphia County Medical Society. January 23, 1890.

Ten Attacks of Appendicitis Within Eleven Months. Operation; Cure. By Eugene P. Bernardy, M. A., Philadelphia. Reprint from Transactions of the Philadelphia County Medical Society. November 13, 1889.

The Popularization of Sanitary Science. Annual address before the Third District Branch of the New York State Medical Association at Syracuse, N. Y., June 19, 1890. By J. G. Orton, M. D., President New York State Medical Association. Reprint from *The Sanitarian*, August, 1890.

Case of Corneal Transplantation from the Rabbit's to the Human Eye, and A Singular Case of Injury. By William F. Smith, M. D., Member of the Heidleberg Ophthalmological Society, etc., etc. Reprint from *The Archives of Ophthalmology.* Vol. XIX, Nos. 2 and 3. 1890.

Suppuration of the Antrum of Highmore. By Morean R. Brown, Professor of Laryngology and Rhinology at the Chicago Polyclinic. Reprint *The New York Medical Journal*, July 19, 1890.

The Relations of Eye-Strain to General Medicine. By George M. Gould, M. D., Ophthalmic Surgeon of the Philadelphia Hospital. Reprint from *The Medical News.* August 23, 1890.

Address in Hygiene. By Thomas J. Mays, M. D., of Philadelphia. Reprint: Transactions of the Medical Society of the State of Pennsylvania. June, 1890,

Proceedings of the First Annual Meeting of the Tri-State Medical Association of Alabama, Georgia, and Tennessee. Held in Chattanooga, Tenn., October 15 and 16, 1889.

Fibro-Myoma of the Uterus. By Edwin Ricketts, M. D., Professor of Gynecology and Abdominal Surgery in the Polyclinic. Reprint: *The Cincinnati Lancet-Clinic.* August 2, 1890.

Diseases of Female Pelvic Organs. When to Operate by Abdominal Section. By M. B. Ward, M. D., Professor of Gynecology, Kansas Medical College, Topeka Kansas. Chicago. Reprint : *The Journal of the American Medical Association.* August 23, 1890.

Time of Conception and Duration of Pregnancy. By Geo. J. Engelmann, M. D., Fellow of the American Gynecological Society, etc., etc. St. Louis : Reprint : *St. Louis Courier of Medicine.* May, 1890.

The Pathology and Treatment of Intra-Pelvic Inflammation. By M. B. Ward, M. D., Professor of Gynecology in the Kansas Medical College, etc., etc. Topeka. Reprint : *Kansas Medical Journal.* August, 1890.

Description of a Series of Tests for the Detection and Determination of Sub-Normal Color-Perception (Color-Blindness). Designed for use in Railway Service. By Charles A. Oliver, M. D. Philadelphia : Reprint : Transactions American Ophthalmological Society. 1888.

An Explanation of the Phenomena of Immunity and Contagion Based upon the Action of Physical and Biological Laws. By J. W, McLaughlin, M. D.; Austin, Texas. Reprint : Transactions Tri-State Medical Association.

The Study of Bacteriology in Medicine. By Frank T. Billings. Paper. pp. 20.

Spinal Surgery. A Report of Eight Cases. By Robert Abbé, M. D., Surgeon to St. Luke's Hospital, New York ; Professor of Surgery, Post Graduate School, etc. New York. Reprint : *Medical Record,* July 26, 1890.

HEALTH BULLETINS.

Newport, R. I., August and September, 1890.

Michigan State Board of Health, August and September, 1890.

Monthly Bulletin, New York State Board of Health, July and August, 1890.

Abstract of Sanitary Reports, U. S. Marine Hospital Bureau, Washington, D. C. Vol. V., Nos. 35, 36, 37, 38, 39, 40, 41, 42 and 43.

---

### NOTICE TO CONTRIBUTORS.

We are glad to receive contributions from every one who knows anything of interest to the profession. Articles designed for publication in the JOURNAL should be handed in before the first day of the month.

The Editors are not responsible for the views or opinions of contributors.

All communications should be addressed to

284 FRANKLIN STREET, BUFFALO, N. Y.

